# Towards Electrochemical Water Desalination Techniques: A Review on Capacitive Deionization, Membrane Capacitive Deionization and Flow Capacitive Deionization

**DOI:** 10.3390/membranes10050096

**Published:** 2020-05-12

**Authors:** Gbenro Folaranmi, Mikhael Bechelany, Philippe Sistat, Marc Cretin, Francois Zaviska

**Affiliations:** Institut Européen des Membranes, IEM, UMR-5635, University Montpellier, ENSCM, CNRS, Place Eugène Bataillon, CEDEX 05, 34095 Montpellier, France; gbenro.folaranmi@etu.umontpellier.fr (G.F.); philippe.sistat@umontpellier.fr (P.S.); marc.cretin@umontpellier.fr (M.C.)

**Keywords:** brackish water desalination, electro-sorption, carbonaceous electrode materials, process consideration, capacitive deionization configurations

## Abstract

Electrochemical water desalination has been a major research area since the 1960s with the development of capacitive deionization technique. For the latter, its modus operandi lies in temporary salt ion adsorption when a simple potential difference (1.0–1.4 V) of about 1.2 V is supplied to the system to temporarily create an electric field that drives the ions to their different polarized poles and subsequently desorb these solvated ions when potential is switched off. Capacitive deionization targets/extracts the solutes instead of the solvent and thus consumes less energy and is highly effective for brackish water. This paper reviews Capacitive Deionization (mechanism of operation, sustainability, optimization processes, and shortcomings) with extension to its counterparts (Membrane Capacitive Deionization and Flow Capacitive Deionization).

## 1. Introduction

The looming and upsurge of unavailable/insufficient freshwater resources is one of the major problems confronting humans and there is no denying that human activities and unprecedented environmental disasters are a contributing factor to this. 

In total, 98% of the water present on Earth is either sea or brackish water which is not readily available for direct use by humans [1]. There are various methods of water desalination such as reverse osmosis (RO), electrodialysis (ED), multi-stage flash distillation (MSFD), multi effect distillation (MED) etc. put in place to circumvent this situation [2]. RO and MSFD contribute most significantly to industrial water desalination from sea water and have been in the front line of major water treatment plants worldwide. Reverse osmosis accounts for most desalination process with 64%. MSFD, MED, and ED account for 23%, 8%, and 4%, respectively [2].

**Figure 1:**
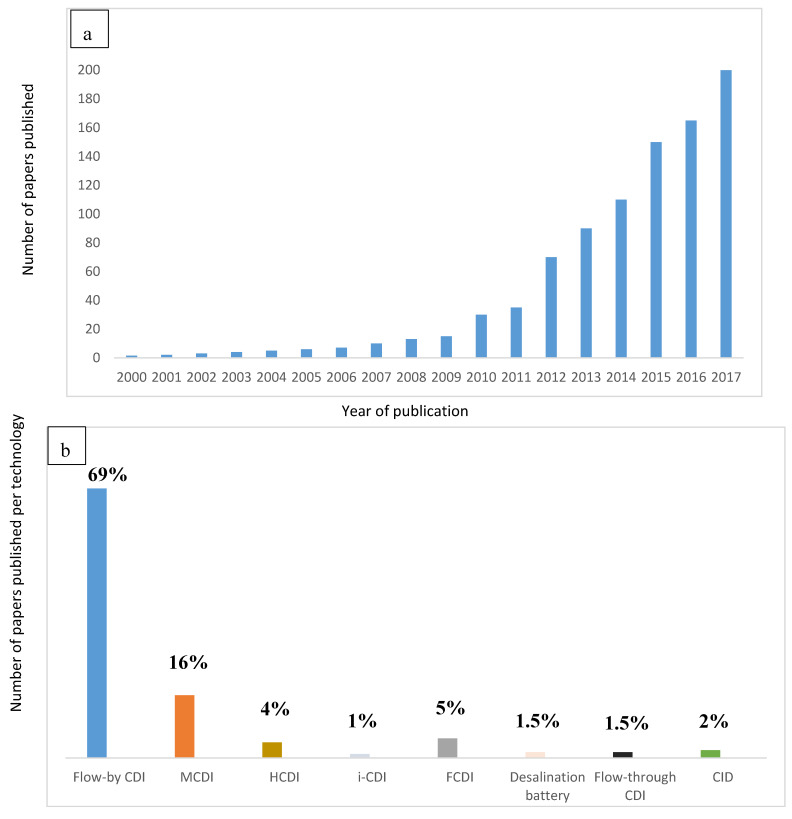
Advances in electrochemical technologies for desalination (**a**) in terms of published papers by Wang et al. [3] (**b**) in terms of exploitation/utilization per technology.

As an alternative to membrane desalination, a new focus of research in which desalination principle is based on capacitive deionization method (CDI) by adsorption process has been developed. Figure 1a shows the advances in publications since 2000. Figure 1b shows the exploitation of different electrochemical technologies. Finally, Table 1 shows the advances in innovation of these technologies. A full historical detail is explained by Wang et al. [3].

CDI, introduced in 1960, has been suggested as a recommended option for brackish water desalination [4]. Using CDI, feed solution is made to pass through oppositely charged electrodes polarized by the application of a potential difference (≤1.4 V) thus causing electric field in the medium and as a result, there is an interface created between electrodes and the electrolyte in which the solvated ions are adsorbed. Subsequently, the created electric field is cut off at zero voltage and the adsorbed ions are released (due to the decomposition of electrical double layer (EDL)) [5]. 

The material aspect of this technology (electrode materials) is one of the key factors or backbone of this electrochemical desalination process. Carbon as a cheap material has served as a precursor for electrode formation due to its availability, porosity, large surface area, amidst other factors. However, it does not possess all the suitable properties needed to make it a standalone material thus leading to myriad of research in new hybrid electrodes development [6]. This review will not focus on the energetic comparison demand between RO and CDI as there are controversial conclusions regarding this aspect of the two processes but highlight some recent improvements and trend in electro-sorption processes. In Section 1, we will give a brief oversight into RO and ED. The proof of concept of RO and ED desalination methods will be highlighted. In Section 2, capacitive deionization systems will be introduced focusing first on capacitive deionization with the aim to link performance (production and quality) and cost (OPEX and CAPEX) with operating parameters, electrode materials, and processes. The main performance indicators will be described to go further in the description of capacitive systems. The limitations of basic capacitive deionization, membrane capacitive deionization and flow capacitive deionization will be reviewed in Section 4 and Section 5. Hence, challenges and future perspectives in desalination will be proposed to conclude this review. 

## 2. A Brief Oversight into Reverse Osmosis (RO) and Electrodialysis (ED)

In this section, the operation mode, energy utilization, advantages, and shortcomings of reverse osmosis and electrodialysis are briefly highlighted.

### 2.1. Reverse Osmosis

This is a membrane method of sea water purification process using pressure as a driving force to make feed solution pass through a membrane usually semi-permeable in nature. During this process, the ions in the feed solution are trapped by the membrane as they move through it. Basically, the sea water is fed into the system and made to permeate the semi-permeable membrane under pressure and recovered as clean water. This is then followed by several post-treatment methods like disinfection, pH, and mineralization adjustment etc. to make it suitable for drinking [7]. In RO, as the ions are trapped by the membrane, there is tendency for generation of concentrate (brine solution) formation. 

#### Trend in Energy Consumption of Reverse Osmosis

Sea water desalination is energy intensive whatever the technique employed. Indeed, in a thermodynamic point of view, sea desalination consists of the generation of two streams (concentrate and permeate) with a very high salinity difference corresponding to high chemical potential difference and thus requires a high amount of energy. Concerning sea water reverse osmosis SWRO, the energy consumption is affected to the work of the high-pressure pump, the aim of which is to drive water through a semi-permeable membrane. High pressure is required in order to overcome the osmotic pressure and also to compensate energy loses due to viscous friction in the membrane module (pressure drop) which transforms mechanical energy into thermal energy. 

The energy consumption of SWRO has tremendously decreased in these last decades, passing from 15 kWh m^−3^ in the early seventies to less than 2 kWh m^−3^ nowadays (considering exclusively RO step) [8,9]. This energy decrease is attributed to both membrane materials improvement (membrane with a very good compromise between selectivity and permeability) and process aspect (operating and energy recovery device implementation). Energy recovery devices have now been configured to convert the mechanical energy present in the brine of RO systems back to the high pressure pumps used in treating the water. Nowadays, the specific energy consumption (SEC) of sea water RO (SWRO) including both pre-treatment and post-treatment is around 3.5–4.5 kWh m^−3^. 

Hence, RO is actually the most developed technology for sea water desalination which is due to the tremendous improvement of membrane material and process optimization for minimizing energy consumption. As showed in Table 2, the specific energy consumption for high capacity SWRO plants is in the range of 3.5 to 6.7 kWhm^−3^ which confirm the important energy consumption reduction.

Membrane properties (mechanical, biological, and chemical strength) play important roles in the viability of RO performance and research has been geared towards optimizing these properties having been an integral part of RO technology. 

Actually, the best SW desalination plant around the world operate RO system below 2 kWh/m^3^ which is becoming close to the thermodynamic limit (1.1 kWh/m^3^ at 50% water recovery from sea water) and even closer to the minimal practical energy consumption (1.54 kWhm^−3^ Elimelech et al. [3]). However, as any other membrane process, RO is very sensitive to membrane fouling and thus requires strong pretreatment for assuring long term filtration operation. While RO operation costs are directly linked to the feed water salinity, the pre-treatment cost is more or less constant even for low salinity water. From this point of view, RO is not the most suitable desalination technology for moderate and low salinity water (below 5 g/L). Electrochemical technology such as ED and CDI seems much more appropriate for moderate and low salinity water as the functioning principle is at the opposite of the others desalination method by targeting solute (ions) instead of the solvent. 

In a comparative energetic study by Biesheval et al. [17], using a series of 13 stacks membrane capacitive deionization (MCDI) cells and feed salinity of NaCl ranging from 0.4–5.2 g/L and RO of 1.7–7.0 g/L feed salinity, it was found that the majority of RO system shows energy consumption less than 2 kWh.m^−3^ while with MCDI, feed salinity below 3 g/L could be advantageous over RO. Their result proves that MCDI can be competitive with RO only at feed salinity below 2 g/L.

### 2.2. Electrodialysis 

**Figure 2:**
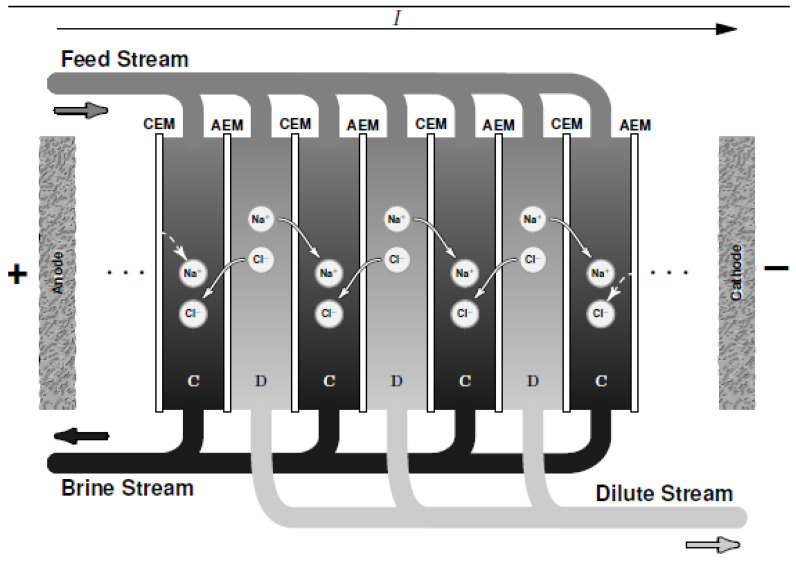
A schematic diagram of electrodialysis (ED) where C is the concentrate compartment and D is the dilute compartment.

ED is a membrane-based desalination method for brackish water. Contrary to other desalination technologies, ED as shown in Figure 2 employs a difference of electrical potential (as well as CDI) as a driving force in order to extract charged compound (instead of the solvent) from a saline solution. It involves ions migration through intercalation of anion and cation-exchange membranes when an electric field is created by difference of potential. Cation- and anion-exchange membranes (CEM and AEM) are alternatively oriented between the anode and the cathode. The application of potential difference in the system electrostatically attracts the cations to the cathode (negative pole) and anions towards the anode (positive pole). Concentrate is generated (brine solution) in ED compartments due to the retention of ions by opposite membranes and consequently fresh water is generated by another compartment like ions migrate through the desired membranes [18]. As shown in Figure 2, a unit of ED is made of an arrangement of CEM and AEM defining the concentrate and the dilute compartments. Improvement studies for performance prediction and optimization such as mathematical models i.e., Stefan–Maxwell theory [19] and Nernst-Planck equation [20] have been employed. For more details, readers can refer to the review on ED proposed by Amshawee et al. [21].

#### Trend in Energy Consumption and Shortcomings of Electrodialysis 

ED has been used in cooperation with other renewable energies such as solar and wind [22] to improve efficiency and reduce energy consumption. ED has also been used coupled with photovoltaic (PV). The integration is environmentally friendly as no pollution is recorded and offers low maintenance cost [23,24]. Economically, hybrid ED form is more expensive than the conventional one. Conventional ED for brackish water treatment costs 0.45–0.78 € m^−3^, while PV-ED costs 6.34–11.93 € m^−3^ [24].

In general, ED is advantageous by affording high removal salt rate with water salinity less than 5 g L^−1^ and energy consumption of 0.8–1.5 kWh m^−3^ at a water productivity of 45 L m^−2^ h^−1^ [25]. It is noteworthy to keep in mind that both ED and CDI are not intended to compete with RO but are more interesting (i.e., in terms of energy consideration) for saline water treatment. ED offers the advantage (unlike other desalination techniques) of being flexible whether in terms of demineralization rate or recovery rates. In addition, CDI and ED are much less sensitive to clogging and therefore require less extensive pre-treatment and therefore are more applicable for the treatment of saline water.

## 3. Capacitive Deionization Systems

Similar to ED, CDI is closely related to ED as it uses electrochemical water desalination technology for desalting brackish water. CDI selectively targets solutes in water making it energetically effective for moderate and low saline water. Unlike ED that operates by intercalation of IEMs for ions exclusion, CDI makes use of porous carbonaceous materials as solid electrodes in temporary storage of ions and the fact that it makes use of porous materials gives it an edge and enviable diversity in terms of functionalities and other applications over ED. CDI operates under low potential difference to polarize electrodes and as a result, the ions get separated by electrostatic attraction to oppositely charged poles, and stored/adsorbed in the pores of the electrodes. 

When an external direct voltage (usually below 1.4 V) is applied onto a system containing saline solution (feed solution), the electric field created electrostatically drives the solvated ions in the solution into their different polarized poles where they are adsorbed in the pores of the electrodes and the process continues until the pores are saturated. Conversely, as soon as the external electric field is removed (short circuiting), the ions are desorbed from the pores of the electrodes (generating concentrate) hence leading to regeneration of saturated electrode materials. 

The fact that the electrodes can store and easily dissipate charges when needed make them qualified as capacitors. When the charges are stored via a non-faradaic process (no redox peak observed), the capacitor is considered as a pseudo or an electrical double layer (EDL) capacitor while it is considered as a super-capacitor if the carbon material is in combination with transition metal oxides (TMOs) and charges are stored under faradaic condition making adsorption process occurs via redox reaction. Supercapacitors possess a high capacitive ability but the performances are limited by water electrolysis at high voltage (>1.4 V) leading to undesirable by-products and energy loss. Veronica et al. [26] did a thorough review on pseudo-capacitors for electrochemical energy storage. 

From an experimental point of view, capacitance derivation could be evaluated via cyclic voltammetry (CV). In the absence of faradaic contributions and in galvanostatic mode, capacitance can be obtained directly from the voltammogram recorded at different sweep rate:

(1) C=ᶘ dqdV=I ᶘdtdV=Iv

where q = charge (C), V = potential difference (V), t = time (s), I = intensity (A), and v = sweep rate (Vs^−1^). The behavior of electrode materials towards electro-adsorption could be directly obtained from resulting voltammograms.

### 3.1. Principle of Ion Adsorption

**Figure 3:**
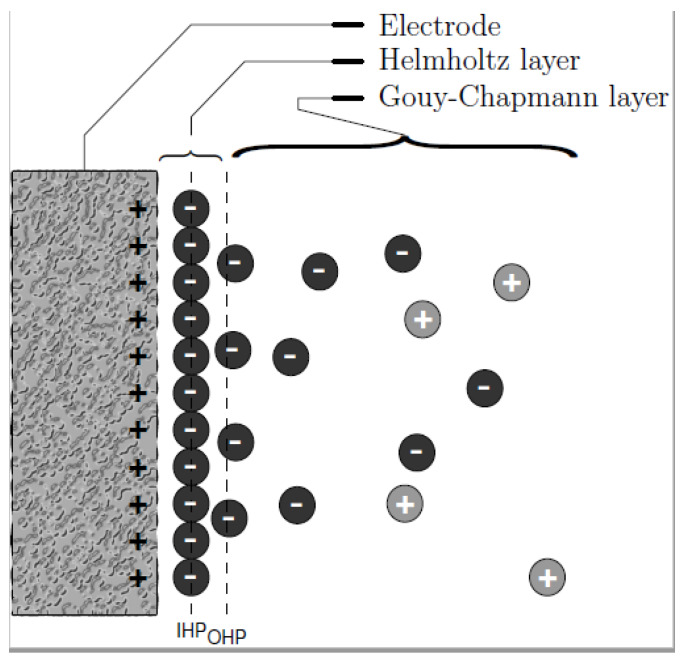
Distribution of charges as described by Gouy–Chapman.

The phenomenon of ions adsorption lies on the EDL created when an accumulation of ions is formed at the electrode–electrolyte interface due to the electrical potential. As proposed by Helmholtz in 1883 [27] in capacitors (Figure 3), distribution of charges at the double layer is governed by charge surface accumulation of one sign while the opposite charges are accumulated at the solution side. Based on the assumption of Gouy-Chapman-Stern model, the double layer can be divided into an ‘inner’ region and a ‘diffusion’ region. The inner region is referred to as the Helmholtz layer where ions covered directly onto the surface of electrode while the region farther from the surface is a diffusion layer called the Gouy–Chapman layer [28] in which the distribution of electric charge depends on the potential at the surface. 

### 3.2. How Does Capacitive Deionization (CDI) Work?

**Figure 4:**
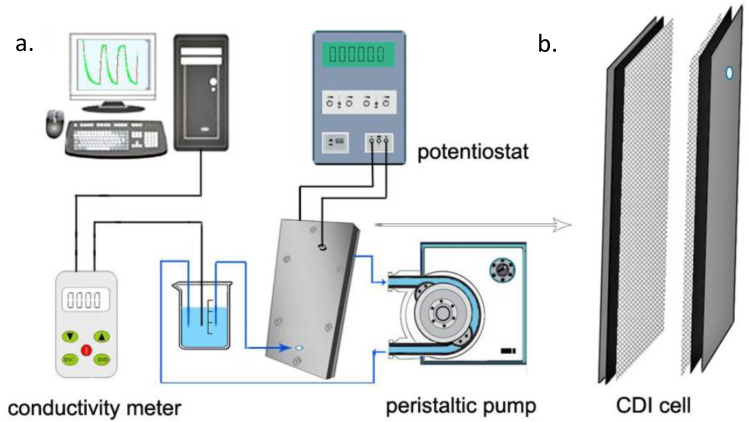
Schematic diagram (**a**) of capacitive deionization CDI set up [29], (**b**) CDI cell.

Simply, a saline feed solution is made to flow by a pair of electrodes that are separated by a spacer. For pseudo-capacitors, no redox reaction occurs (absence of Faradaic reaction) and the response is purely capacitive in nature. Potential difference is generated by an external source to the system enabling electrostatic attraction of ions to the oppositely charged electrodes (adsorbed) until the pores of the electrodes are saturated. Consequently, desorption occurs by the desorption of adsorbed ions which is made evident by the increase in ionic conductivity that is being monitored by a conductimeter. Figure 4a shows a schematic laboratory CDI set up in which the potentiostat powers the cell and the current response in relation to electrosorption is made evident by the monitor of a computer that is connected to the potentiostat. The influent solution is fed in by a peristatic pump at a constant flow rate and the ionic conductivity change is monitored and recorded by a conductimeter. Figure 4b shows a laboratory CDI cell; it consisted of two parallel electrode sheets separated by a non-electrically conductive spacer of about 500 to 1200 microns thick. This prevents an electrical shortage and allows liquid to flow 

**Figure 5:**
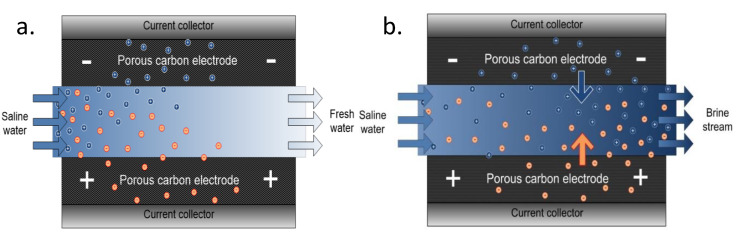
Schematic representation of CDI during (**a**) adsorption and (**b**) desorption process.

Schematic representation of fresh water and brine generation during adsorption and desorption processes is shown in Figure 5a, and Figure 5b respectively. In adsorption process as shown in Figure 5a, the solvated ions are electrostatically attracted to different polarized poles (electrodes) as a result of electric field created thus producing fresh water while in desorption process; Figure 5b, brine generation is produced due to the release of the ions from the pores of the electrodes once the electric field is cut off at zero volt. CDI is a low-pressure deionization technique that does not require membrane. CDI however has a major challenge of co-ion adsorption as there is no means to screen off oppositely charged ions thus leading to low charge efficiency of the system. There is then a great interest to work on electrode materials to increase overall performance of the system. Additionally, it is an intermittent process—there is a lag of time as adsorption process is limited to the fixed and immobilized electrodes on the current collectors thus limiting adsorption rate. Biesheuvel et al. [30] described a thermodynamic model for the CDI charge efficiency improvement i.e., by increasing cell voltage, Stern capacity, or decreasing the ionic strength of the solution being treated. 

## 4. Electrode Materials

One of the key factors influencing the overall efficiency of CDI technology is the material aspect. Undoubtedly, electrode is considered suitable for CDI if conditions such as: high surface area, high porosity, good electronic conductivity, good stability, high capacitance, hydrophilicity, and must be economically feasible for industrial optimization in terms of availability, cost, and recyclability/biodegradability are met. In light of this, carbon-based materials fit into all the properties but none of them single handedly possess all the criteria/properties needed as high performance CDI electrodes hence various methods are employed to influence the properties of carbon-based materials. Some of the improvements in the material aspect as found in literature are discussed below.

### 4.1. Carbon Electrodes with Various Morphologies and Porosities

Various forms of carbon have been exploited, some of which are: activated carbon (AC), activated carbon fiber, carbon aerogel, activated carbon cloth, carbon nanotubes, graphene, carbon nanofibers, carbon sheet, etc. [31,32,33,34,35,36]. In most cases, carbon-based materials are converted to electrodes by turning them to dispersion solution and deposited or immobilized on a support (current collector) via many methods such as spray coating [37], pressure pressing [38], blade coating [39], and through electrophoretic deposition [40]. Some major limiting factors for instance in AC are in terms of wettability, low porosity, and conductivity. In graphene for instance, its intrinsic property of restacking possesses a major drawback for its usage as electrodes. However, in order to improve their efficiencies and other shortcomings, they are often treated both physically and chemically for an efficient ion adsorption capacity. Physical treatment such as thermal application modification under inert gases (N_2_, Ar, CO_2_, etc.) for pores expansion has been employed. Depending on the operating conditions during the thermal process, pore volume and microporous or mesoporous regions of activated carbon are usually influenced under this method [41]. Under chemical treatment, a lot of modifications exist depending on the property to be improved. For instance, surface area can be improved by treating with KOH or NaOH after which it is subject to further thermal treatment [42]. With respect to wettability, the introduction of oxygenated functional groups (OFGs) into the surface of activated carbon (increase of surface hydrophilicity) is done as reported in literature through oxidation by acid (i.e., nitric acid) [43] or oxidizing agents like potassium permanganate (KMnO_4_) or hydrogen peroxide (H_2_O_2_) [44,45].

### 4.2. Hybrid Carbon-Based Electrodes

Hybrid composites of activated carbon with materials of higher performance have been reported in literature [46,47,48]. Transition metal oxides (TMOs) for instance have been used by many researchers to enhance carbon-based electrode stability, conductivity, and electrochemical performance. Advanced chemical methods like chemical vapor deposition (CVD), ice segregation isolated self-assembly (ISISA), and electrospinning [49] are also being exploited to make 3D carbon of high porosity and conductivity.

### 4.3. Alternative Carbon Source-Based Electrodes

It is of interest to know that the intrinsic properties of carbon materials are based on their methods of preparation and precursors used. Of recent, carbon derived carbide (CDC) [50] and metal organic framework (MOF) [51] as sacrificial precursors as well as carbon monoliths [52] have drawn the attention of researchers in CDI. They possess suitable characteristics needed for electrodes i.e., high surface area, porosity, and wettability. The performance of resorcinol-based mesoporous carbon (MC) in comparison to that of carbon aerogel (CA) was studied by Tsouris et al. [53] using feed salinity concentration of 1000 ppm and 35,000 ppm, their findings showed that resorcinol-based MC (adsorption capacity of 15.2 mg/g) has higher adsorption capacity over CA (adsorption capacity of 5.8 mg/g). It was suggested that the polymer precursor and the method of synthesis employed conferred higher porosity on MC hence improving adsorption. 

Alternative carbon source of high performance based on sacrificial materials i.e., carbon derived carbide (CDC) has attracted attention. CDC reported by Arulepp et al. [54] showed excellent volumetric capacitance of 90 F cm^−3^ for non-aqueous EDL capacitors over commercial AC. AC derived from Fe-MOF in combination with grapheme oxide when investigated by Wenhua et al. [55] generated a highly porous and electrical conducting carbon network enabling higher electrosorption (37.7 mg/g at feed salinity of 1000 mg/L NaCl). 

Precursory sacrificial MOFs for AC synthesis usually yields AC of high porosity and specific surface area though this depends on the type of crystallinity and porosity of the MOF used [56].

### 4.4. Carbon Electrodes Modified by Nitrogen Doping

The introduction of nitrogen into carbon i.e., nitrogen doped graphene (NG) has been reported in literature mainly due to the improvement of electrical conductivity and wettability of the interface between the electrolyte and the electrodes thus facilitating electron transfer and ion transport as well as increase in specific surface area [57,58]. As further reported, NG tremendously recorded an improved specific capacitance in the range from 140 to 326 F g^−1^, much higher than that of pristine graphene (PG) (135 F g^−1^) [59]. Yang et al. [60] improved the cyclability rate of potassium ion battery by intercalation of nitrogen into carbon nanofibers. 

An abundant literature is devoted to this subject but from our point of view, graphene nitrogen doped carbon, MOF derivate carbon, as well as CNT are the most promising materials in the field. The literature described above and references given in Table 3 below will help the reader get a good understanding of the problem for the best choice of material electrodes. 

### 4.5. Carbon-Based Electrodes in Alternatives Applications

Apart from basic desalination application, hybrid carbon-based electrodes have been successfully exploited in environmental studies. Qinghua et al. [71] using pyridinic N-dominated graphene (N-6-G) and pyrrolic N-dominated graphene (N-5-G) were able to capture lead (Pb^2+^) in aqueous solution, thus opening way for selective desalination in complex media. With the same approach, arsenic-contaminated groundwater in Taiwan was studied by Chen et al. [72]. Carbon aerogels has been shown to remove various inorganic species from aqueous solutions by Christophe et al. [73]. Bao group [74] used resin activated carbon electrode to successfully recovered vanadium from aqueous vanadium solution. 

## 5. CDI Treatment Objective, Performance, and Parameters: Process Considerations

In order to develop an “optimal” cost effective CDI process, it is essential to identify and understand the effect of the main parameters affecting the electrochemical separation process. By the means of “optimal” it refers to both design aspect as well as operating conditions in order to reach a given objective of efficiency, best compromise in term of capital cost (CAPEX) and operational cost (OPEX). Also, it is essential to define the performance criteria of a CDI system which is briefly discussed below.

### 5.1. Criteria of CDI Performance Evaluation

#### 5.1.1. Maximum Salt Adsorption Capacity (mSAC)

The concept of this parameter was firstly introduced in 1972 by Sofer and Folman [41]. It is related to the mass of ions adsorbed as a function of adsorbent weight. The value of mSAC is expressed in mg of salt adsorbed per g of electrode material and it is calculated using Equation (2):

(2) $$mSAC=\int_{0}^{tads}\left(Ci-Cf\;\right)ᶲv\;dt$$

where ᶲv is the flow rate of solution in the CDI circuit, C_i_ is the initial concentration of the solute, C_f_ is the final solute concentration, and t_ads_ is the duration of the adsorption step. The maximum SAC is typically reported as the ratio of maximum weight of salt removal to the total weight of adsorbent, expressed in mg/g of electrodes where mg is the amount of salt adsorbed, g is the weight of the adsorbent. It is important to note that the mSAC depends mainly on the intrinsic electrode properties (pore size, surface area, capacitance, surface physico-chemical properties etc.) but also on design consideration (i.e., electrode thickness) and on the electrochemical conditions (voltage, hydrodynamic, feed salinity, etc.). The mSAC can be determined from the evolution of the conductivity, when its variation in function of time becomes negligible. However, as long as the adsorption phase is running, kinetics of salt adsorption becomes lower until reaching the equilibrium. In other word, in a practical point of view, it is not pertinent to reach the mSAC which will require a hydraulic retention time (HRT) that is too high, making the CDI process not favorable in terms of CAPEX. 

In summary, mSAC is related to the storage capacity and represents one of the most important parameters for evaluating the performance of an electrode. Considering that CDI is an intermittent process alternating adsorption and desorption phases, the mSAC will directly determine the frequency of changing phases and will impact both CAPEX and OPEX. 

For practicality and a general view of mSAC as an indicator index, we could consider that the typical value of mSAC often used as a reference is the one obtained from the AC electrode and it ranges between 3–5 mg/g. Such mSAC value is a bit too low for industrial application. mSAC values above 15 mg/g seems appropriate for CDI application [49,50,51,52].

#### 5.1.2. Average Salt Adsorption Rate (ASAR)

Average Salt Adsorption Rate (ASAR) is a parameter related to the kinetics of the adsorption process, in other words, how fast ions can be adsorbed onto the electrode per unit of time of the adsorption process. It is defined as:

(3) $$\frac{Q}{\mathrm{NAtads}}\int_{0}^{\mathrm{tads}}\left(\mathrm{Ci}-\mathrm{Cf}\;\right)\;dt$$

The ASAR is expressed in mg/g/min where “min” is the average time in minute for the adsorption or desorption step. Unlike mSAC, ASAR depends on multiple external factors such as flow rate, applied potential, initial feed water concentration, and the cell architecture. Depending on the objective of treatment, there is a compromise to find in order to minimize the alternation of the adsorption/desorption phases (mSAC) and the ASAR. The most important parameter affecting ASAR is the driving force (difference of potential) but other parameters such as the physico-chemical properties, electrode design, as well as the operating conditions (hydrodynamics, feed solution salinity, etc.) affect desalination kinetics. There is no general reference value to assign for ASAR as it is dependent on factors like feed salinity, product salinity, etc.

#### 5.1.3. Current Efficiency (CE)

The term charge efficiency or current efficiency (CE) was used the first time by Avraham in 2009 [75]. The charging efficiency of the system also called faradaic efficiency, is the ratio between the electrical charge that serve specifically to adsorbed ions on the total electrical charge applied between electrodes. In other words, CE corresponds to the fraction of current that was really used for desalination. The other fraction corresponds to the ohmic losses and the current associated to the possible faradaic reactions if the voltage exceeds the potential of water hydrolysis (~1.4 V). CE is defined as follow:

(4) $$CE=\frac{I_{\min}}{I}=\frac{z\;\times \;F\;\times \;\left(\mathrm{Ci}\;–\;\mathrm{Cf}\right)\;\times \;\mathrm{Vs}}{M\;\times \;⟆Idt\;}$$

where I_min_ is the minimum theoretical current necessary to remove a given amount of ions (in Amperes), I is the actual applied current, z is the elementary charge on ion, F is the number of Faraday constant (96485 C), C_i_ is the initial molar concentration of the solute, C_f_ is the final solute concentration, Vs is the volume of the solution, M is the molar mass of the solute, while ⟆Idt is the recorded integral current with respect to time. Current efficiency is an important parameter for evaluating the performance of the CDI system. The higher charge efficiency leads to lower energy consumption. The maximum possible efficiency is the one which would occur when one ion is removed for each unit of charge deposited on the electrodes [30,38]. Current efficiencies higher than 80% are desirable in order to minimize energy costs. Low current efficiencies indicate water splitting in the diluent or concentrate streams, or back-diffusion of ions from the concentrate to the diluent.

#### 5.1.4. Specific Energy Consumption (SEC)

Energy consumption is one of the biggest hurdles desalination faces. Nowadays, in a worldwide energy crisis context, it seems not pertinent to talk about desalination performances without taking energy consumption into consideration.

SEC is often used to characterize the energy cost of a desalination process. SEC is expressed in kWh/m^3^ and corresponds to the energy needed to produce one cubic meter of permeate at a desired water recovery. SEC in electrochemical processes can be expressed as the overall electrical power spent relative to one cubic meter produced.

(5) $$\mathrm{SEC}\;=\frac{W_{\mathrm{CDI}}}{V_{\mathrm{perm}.}}=\frac{\int^{}{Ƥ}_{\left(t\right)}.\mathrm{dt}}{V_{\mathrm{perm}.}}$$

where W_CDI_ is the energy supplied, Ƥ is the applied power, t is the time of treatment, and V_perm_. is the volume of desalinated water. The CDI process works usually at constant electrical potential (to avoid parasitic faradaic reaction), letting the current varying over time with the degree of demineralization. Equation (5) can then be written as follows:

(6) $$\mathrm{SEC}=\frac{U.\int^{}I\left(t\right).\;\mathrm{dt}}{V_{\mathrm{perm}.}}$$

where I(t) is the actual applied current (amperes) at a given time, U is the electrical potential, and t is the time of treatment. SEC is also related to CE, Equation (7).

(7) $$\mathrm{SEC}=\frac{U.\int^{}I_{\min}\left(t\right).\mathrm{dt}}{\mathrm{CE}.{\;V}_{\mathrm{perm}.}}$$

However, because CDI (as well as ED) are focusing/targeting solute instead of the solvent, it seems more pertinent to expressed energy consumption on kWh/kg of salt removed instead of kWh/m^3^. Indeed, in comparison to the other desalination processes, electro separation processes can modulate the desalination rate in function of the treatment efficiency. Just like ASAR, SEC is also dependent on factors like feed salinity, etc.

#### 5.1.5. Electrode Stability (STAB)

Stable recyclability of a CDI electrode reveals the uncompromised electrode–electrolyte interaction over a long period of time which is critical for salt adsorption capacity. Electrosorption mechanism has to do with ion adsorption and desorption in which the latter leads to electrode regeneration. Various reports have been found in literature in improving electrode stability by incorporation of transition metal oxides to carbon-based materials [35,76] i.e., graphene-doped MnO_2_ showed excellent recycling stability [35], ZnO doped AC shows stable electrode cyclability [76] and addition of carbon nanotube to ordered mesoporous carbon exhibited high reversibility and electrochemical stability during the charge/discharge process [77]. 

**Figure 6:**
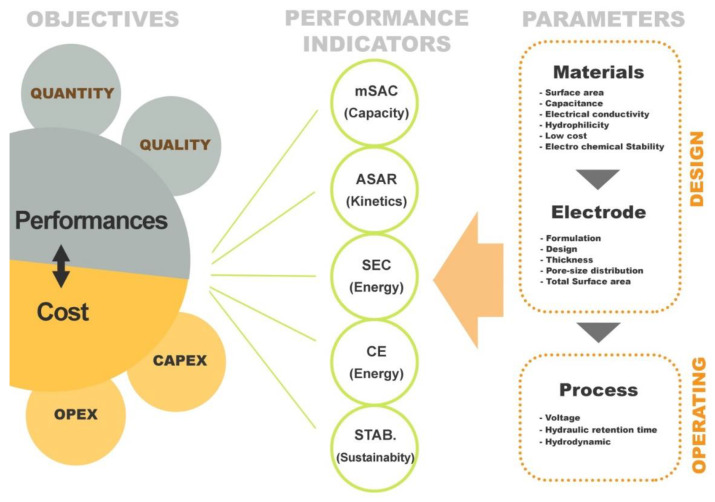
Schematic diagram of CDI performance indices.

Figure 6 summarizes the performance indicators related to a typical CDI process. Several parameters/variables affect the performance of the process from either design or operating point of view. It is important to understand the way of how these parameters affect the performance criteria and how to control/optimize them in order to develop and operate a cost effective desalination process based on electro-sorption. In a first instance, it is necessary to develop/select a good (performant) electrode material as it is at the heart of this technology (e.g., paragraph 3). Then electrode has to be shaped considering formulation (material, conductive additive, binder…), fabrication method, and sizing aspect (thickness, surface area…). Finally, in addition to the design aspect, operate the process at its best condition is also essential to satisfy the most the treatment objectives. Treatment objective for water treatment processes refer to performance in terms of productivity (volume or flow rate) and quality (feed and product salinity) as well as investment and operating cost (CAPEX and OPEX).

### 5.2. Some CDI Operating Process Performance Parameters

The three main operating parameter affecting CDI process are the voltage (or the difference of electrical potential), the hydraulic retention time (HRT), and the hydrodynamics conditions. 

#### 5.2.1. Difference of Electrical Potential

In CDI process, the difference of electrical potential represents the driving force that moves ions from the bulk to the surface of the electrode (EDL). Hence, the higher the voltage, the faster the adsorption is. However, high voltage can cause faradaic reactions such as water hydrolysis which are undesirable for desalination application and will decrease CE. For instance, utilization of voltage above 1.4 V can lead to water splitting and the formation of undesirable by-products which can change the solution pH and leads to electrode oxidation. Basically, salt removal is achieved with increasing potential in the range of 0.8–1.4 V [39].

#### 5.2.2. Hydraulic Retention Time (HRT)

Hydraulic retention time (HRT) is the residence time of feed solution (time spent by the influent inside the cell) which is for a given desalination objective (salt removal and production), purely related to design aspect (sizing). HRT can be calculated by the volume of the cell (exclusively for water circulation) divided by the flow rate. The higher the HRT, the higher the salt removal will be (in conditions that the electrodes are not saturated). 

#### 5.2.3. Hydrodynamics

The flow configuration selected for a CDI system and the hydrodynamic condition associated are important parameters affecting the desalination performances due to their impact on ions transport from the bulk to the electrode surface. Mass transfer of ions from the feed solution onto the surface of the electrode is governed by electromigration, diffusion, and advection phenomena. 

Considering the liquid/electrode interface with one dimensional analysis, three regions can be identified: the bulk or the middle channel, the porous electrode, and the stagnant diffusion layer (SDL) [78]. The thickness of the SDL also called Nernst layer is dependent on the convective mixing and turbulence of the bulk stream which affect the ions transport to/from the electrode surface during the adsorption and desorption phases. Biesheuvel and Bazant give a good description of the ions transport at the liquid/porous electrode interface [79].

### 5.3. Cell Geometries of CDI

Cell geometry/configuration is one of the many process factors that cannot be undermined in CDI aspect. In light of this, different geometries have been improved and the two most conventional types of these geometries are highlighted below.

#### 5.3.1. Flow-by CDI

In the conventional flow-by system, a small planar gap is left in between the electrodes (separated by a separator layer) through which water can flow along the electrodes placed parallel to one another. It affords high energy recovery and low energy consumption. The limitation of this design is that it can only desalinate moderate brackish water and it requires long time of operation. In flow-by system, separator must be carefully optimized to minimize cell electrical resistance and volume while allowing sufficiently large area for efficient fluid flow [80].

#### 5.3.2. Flow-through CDI

In contrast to flow-by CDI, instead of the feed water flowing in-between the parallel electrodes, it is possible to direct it straight through the porous electrodes and parallel to the applied electric field direction, this cell is named flow-through CDI. In this design the feed water is pumped perpendicular to the layered structure that is, straight through the larger pores in the electrodes. This system design offers increase in the kinetics of adsorption by promoting turbulence inside the pores canals and forcing ions to enter in contact with the charged electrode [80]. Major pros and cons of the two geometries are presented in Table 4.

As reflected in Table 3 properties and performance of both flow-through and flow-by CDI geometries are related in part to the development of materials to achieve at the same time high capacity and kinetics for efficient desalination. 

## 6. Membrane Capacitive Deionization (MCDI)

**Figure 7:**
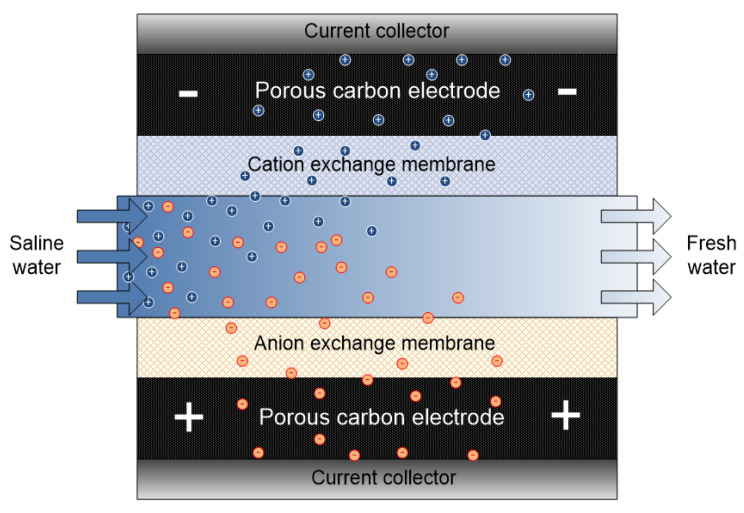
Schematic representation of membrane capacitive deionization (MCDI) system.

The inherent challenge of CDI (i.e., ion co-adsorption yielding low desalting efficiency) led to MCDI development. Conventionally, capacitive deionization techniques (CDI, MCDI and flow capacitive deionization FCDI) operate via a similar mode or mechanism. The major difference comes in the cell configuration/set up. In the case of MCDI, a cation-exchange membrane (CEM) and an anion-exchange membrane (AEM) are placed in front of the cathode and anode respectively as shown in Figure 7. IEM provides screening shield to the exclusion of undesired ions towards the electrodes thereby limiting co-ion adsorption to a great extent. MCDI significantly increases the efficiency of CDI by improving parameters such as charge efficiency, ion adsorption etc. However selectivity, which is linked to material, is compromised.

MCDI has been reportedly used for environmental studies i.e., Wang et al. [81] used MCDI for sulfate removal. Pan et al. [82] investigated the removal of fluoride from water. MCDI has been used in selective removal of nitrate [83] and lithium [84]. As a matter of diversity, Liu group used alternative IEMs (polyethyleneimine and dimethyl diallyl ammonium chloride) casted into carbon nanotube electrodes to achieve 93% efficiency for NaCl removal. A much higher result compared to the 74% efficiency obtained in conventional MCDI [52]. Yoon et al. [85] investigated the use of calcium alginate as a potential IEM. Using this method, higher salt adsorption capacity and charge efficiency was achieved (15.6 mg/g and 95%) as against that of CDI (9.8 mg g^−1^ and 55%). 

Additionally, modification in the flow channel has been reported to improve adsorption capacity in MCDI by deposition of either ion exchange resins [86] or granulated activated carbon [87]. This slight modification leads to a reduction in electrical resistance at low feed salinity with 90% salt removal efficiency [86].

## 7. Flow Capacitive Deionization (FCDI)

The major challenge of CDI lies in its ion co-adsorption. This leads to low charge efficiency and low ion adsorption. By incorporating ion exchange membranes, this challenge was overcome and Membrane Capacitive deionization (MCDI) was developed. Nonetheless, solid electrodes employed in CDI/MCDI allow discontinuous electrosorption process (alternative process of adsorption/desorption phases). Additionally, the static mode of operation (as electrodes are deposited and fixed on a current collector) limits the utilization of availability of pores as electrodes need to be regenerated when saturation level is reached. Additionally, porosity of solid electrode material is compromised as the inclusion of a binder (usually PVDF polymer) drastically reduces available carbon pores and also affects the electrode electrochemical behavior. However, in order to bridge the gap of solid electrode usage and discontinuous mode of operation which apparently cannot be overcome by CDI process hence exploitation in the area of slurry electrodes has been of interest and that led to a new cell design with the name flow capacitive deionization (FCDI). 

**Figure 8:**
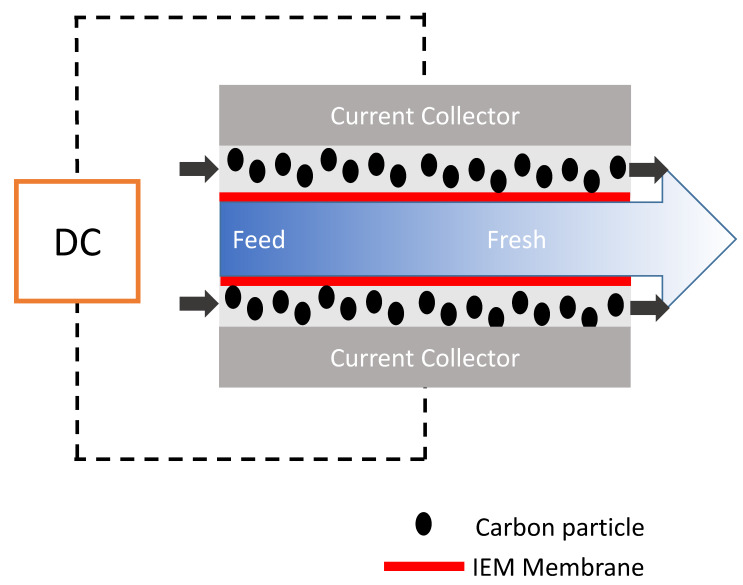
Schematic representation flow capacitive deionization (FCDI) system.

As invented in 2013 indeed, FCDI is based on the slight modification of MCDI by using carbon suspension as flow electrodes [88]. FCDI enables the major benefit relative to conventional CDI in that it affords continuous mode of operation and desalination of high-salinity feed, as shown in Figure 8. In principle, it entails the flowing of suspended carbon electrode (slurry) in a flow path carved on a current collector that is separated between two ion-exchange membranes (CEM and AEM) with salt water running through a spacer. Basically, the guiding principle of operation is the same as that of CDI. The higher the carbon loading (slurry), the higher the adsorption rate as the network of interaction is increased within the active material thus promoting better electron transport. In FCDI, electrical field is created by the passage of voltage from an external source which drives the ions in the feed saline solution to migrate through the ion-exchange membrane, and is eventually adsorbed onto the pores of the suspended carbon slurry electrode. This method proffers a continuous adsorption phase within the cell while desorption takes place outside of the cell (regeneration of the slurry electrode). FCDI, even at high CE and reduced resistance, cannot still compete with RO although it has been successfully proven to be effective for sea water desalination [89].

In terms of comparison, a preferable amount of charge efficiency is improved in solid electrode as there is a high transfer of current by the current collector to the immobilized electrode unlike the slurry form. Indeed the charge is slowly transferred to the flowing carbon slurry due to the limiting major component of the slurry (H_2_O); although at high carbon loading, the carbon slurry electronic percolation or network can be increased to augment this shortcoming but at the expense of viscosity. The consequential effect of increase in the electrode slurry (at high carbon loading) can lead to increase in pressure loss and could lead to clogging of the carbon slurry and also dissipating pumping energy (mechanical) into thermal energy. By modifying cell architecture, only 35 wt. % carbon content of the slurry has been achieved so far [90]. Exploitation of carbon particles of low size (2–10 µm) and spherical carbons as application in slurry electrodes has also been reported thus enabling higher carbon loading and viscosity reduction. The highest percentage of the slurry composition is water which is a poor electrolyte; hence to achieve a network of good electronic conductivity within the active material (carbon slurry), a compact interaction of electron percolation in flow electrode is desired. As found so far in literature, electronic improvement has been achieved with addition of NaCl [91], carbon black (CB) [92], carbon nanotubes [93], and functionalized carbon nanotubes additives (FCNT) [94]. 

Low resistance in flow electrode is a contributory factor in achieving better results in FCDI. Yang et al. [91] studied feed electrode containing high salt concentration and it was revealed that the high resistance created by water (major feed electrode component) was reduced by the addition of 2.44 wt. % of NaCl to the feed electrode hereby enhancing desalination. Effect of CB dosage on internal resistance and high voltage on charge efficiency was also studied by Peng et al. [92]. It was observed that at higher voltage (4.8 V), desalination rate 0.208 mg/(min·cm^2^) was achieved while charge efficiency dropped from 92% to 69.5% due to Faradaic occurrence. Additionally, electrochemical impedance spectroscopy (EIS) revealed that the internal resistance of the FCDI decreased with increased CB dosage.

FCDI stacks has also been studied by Cheol et al. [95] revealing that FCDI with five unit cells shows a higher desalination rate compared to single FCDI unit cell. Using two FCDI modules, Gendel et al. [96] developed a continuous mode of electrosorption process in which 99% desalination rate was achieved and 90% water recovery was reported. A new FCDI without the use of IEMs has been demonstrated by Hatzell et al. [97] to desalt brackish and seawater.

**Figure 9:**
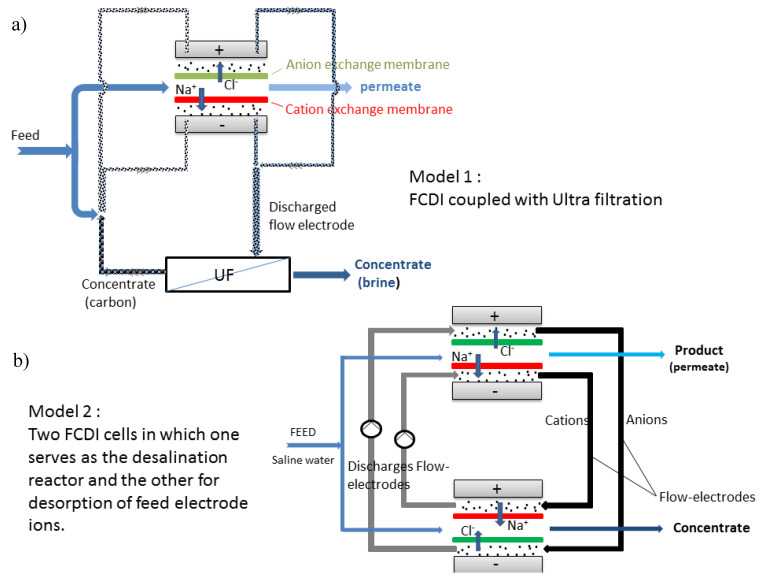
Schematic diagrams of FCDI in continuous mode via (**a**) FCDI coupled with ultrafiltration and (**b**) two FCDI cells.

Most FCDI systems are still in batch mode of operation. Report in the continuous mode is sparse in literature. FCDI continuous module employing two CDI set up offers recyclability of feed electrode. Figure 9a shows a typical module of FCDI in a continuous mode as developed by our group. In Figure 9b, two cells are present in which one serve as a desalination reactor while the other serves as concentrate generator. The possibility of continuous module could offer an upscale in desalination rate with the possibility of energy recovery as present in batch mode. The other possibility of continuous operation is coupling the FCDI cell with ultrafiltration technique in which the feed electrode ions are recycled by ultrafiltration technique. The major drawback of this technique is that energy recovery might not be possible. 

### Energy Recovery in FCDI

This is an aspect that is fast developing. Here, some of the energy used during desalination process can be recovered. Undoubtedly, pumping of slurry in FCDI gives room for possibility of additional energy requirement [98]. Energy recovery up to 83% in CDI [99] and 20% in FCDI [100] have been reported but operated in a discontinuous mode. However, Rommerkershin et al. [101] using continuous FCDI systems was able to recover up to 36% of applied energy. Junjun et al. [102] was able to recover maximum energy ratio of 7.6%. Additionally, Jeon et al. [88] incorporated salt to the flow electrode easing ionic resistance and confirmed electrical energy recovery of about 20%. Studies by Lim et al. [103] confirmed that improving energy capacity in FCDI can be achieved by increasing the capacity of electrode slurry container and that the feed solution at high salt concentration (salt water) is advantageous in terms of higher capacity for both energy storage and generation compared with feed solution of low concentration (brackish water). 

As found in literature, Table 5 summarizes the varied parametric conditions in terms of slurry composition, feed solution composition, and operating conditions in FCDI process. 

## 8. Summary, Challenges, and Future Perspectives in Desalination

There have been numerous studies with the intention of upscaling water desalination technologies. Studies geared towards high water recovery, membrane design for high selectivity, permeability, and less fouling of membrane have been reported. RO application in water desalination has been in existence for over five decades due to its high ability in water recovery [108]. RO acts as a physical barrier against almost every contaminant in water and provides a very good and stable/reliable water quality. It has the ability to reclaim wastewater and desalinate both sea and brackish water because of its utilization of multifunctional membrane materials that offers the highest ions and organic contaminants rejection at a reasonable permeability. Membrane limitations such as membrane fouling and scaling still offer major challenges in this field. With recent advances in energy demand, RO has recorded a great reduction in energy consumption to as low as 2 kWh/m^3^ [109]. Furthermore, RO has been coupled with other processes such as solar photovoltaic system to power RO desalination [110]. In addition, the introduction of new membranes like biomimetic membranes that offer higher permeability, higher resistance to biological and chemical attack has been reported and bring high advances in this field [111]. 

**Figure 10:**
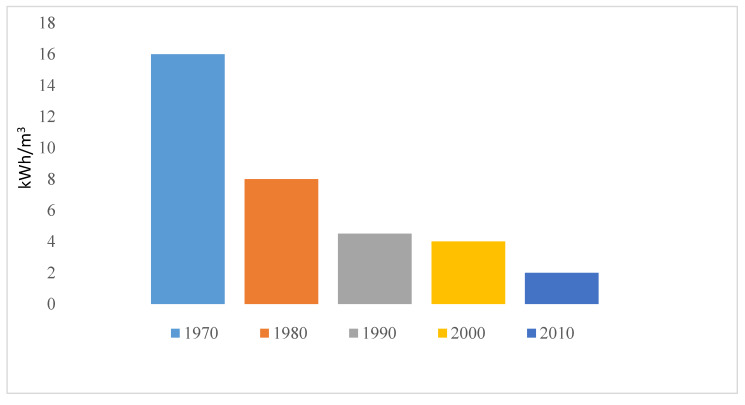
Trend in energy reduction in ED as reported by Sajjad et al. [21].

ED is a membrane-based technology operating on the principle of electro-separation of ions. Advancement in research and technology has led to tremendous decrease in energy consumption (from 16 to 2 KWhm^−3^) of ED as made evident in Figure 10 [21]. ED has been applied for processing municipal wastewater, nutrient recovery, underground brackish water and industrial wastewater (effluent) [21,22]. Introduction of mathematical model to ED has maximized the performance from saline concentration from less than 5000 mg/L to 15,000 mg/L [26]. The limitation of ED still lies in its high investment cost and high energy consumption for salinity above 5 g/L. Pilot scale development of ED for industrial applications as an alternative for RO is currently on going in Singapore [112] for public water supply (Evoqua desalination technology, Singapore). 

CDI is a potential alternative brackish water desalination technology. CDI electrochemically removes ions from salt water by using porous electrodes. A stream of salt water flows in between two electrodes held at a potential difference of around 1.2–1.4 V; as a result of the electric field created, ions in the saline solution are separated, attracted to the oppositely charged electrode where they are adsorbed. This process of deionization (adsorption phase) continues until the electrodes are saturated with ions and then subsequently fed back as brine into another circuit hence, causing regeneration of electrodes (desorption phase). Cell geometries and electrode nature are one of the factors affecting the performance of this technology. Nanomaterials such as graphene and CNTs have been drawing increased attention in this field due to their high specific surface area and electrical conductivity. CDI and MCDI have proven their cost-effectiveness for brackish water treatment, however only few pilot scale demonstrations of CDI or MCDI have been performed so far [113,114].

CDI in general has been promoted as a cheap and arguably low-energy alternative technique to RO. Qin et al. [91] reported the energetic performance of CDI and RO. Their findings show that RO is significantly more energy efficient than CDI particularly when targeting higher salinity feed streams and higher salt rejection values. They argued by rationalizing factors such as feed salinity, average water flux, salt rejection and water recovery affect the energetic of both RO and CDI. Employing CDI to treat feed salinity of 2000 mg/L NaCl, 50% water recovery, 75% salt rejection, and SEC of 0.85 KWhm^−3^ was achieved; a value more than 8 times higher than RO (0.09 KWhm^−3^). According to Voltea (world’s leader in MCDI), the capital cost involved in setting up installation sites of CDI is comparatively lower to that of RO. So, considering the argument posed by Qin et al. [91] and Voltea’s (CapDI-capacitive deionization company), a general consensus cannot be reached as one has to compromise between cost and performance between CDI and RO. In fact, according to the study conducted by Biesheval et al. [17], MCDI with feed salinity below 3 g/L NaCl could be advantageous over RO in terms of energy consumption. Additionally, fouling and scaling of membranes are contributory factors that might lead to increase in energy consumption. Although introduction of ion-exchange membranes can significantly improve efficiency by expelling co-ion adsorption phenomenon in CDI; however, selectivity could be hindered. The cost of membranes can also be reduced by alternatively employing light conducting cheap membranes. This is believed to drastically reduce membrane fouling caused by natural organic matter [115]. A thorough review on roles of membranes in MCDI was reported by Armineh et al. [116].

Suitable materials that can qualify as ideal electrodes while operating at a non-Faradaic condition has been a focus of interest [117,118]. Non-Faradaic reaction in CDI is always encouraged because Faradaic CDI process (voltage above 1.4 V) leads to carbon electrode deterioration, pH fluctuation, production of byproducts and water splitting, and subsequently increases energy consumption [119].

A major issue relating to material aspect is cost. Electrode materials possessing long term stability, high electrical conductivity characteristics, and good regeneration rates are expensive. In order to minimize cost, vigorous research should be channeled more in optimizing cheap materials such as AC. CDI is a low pressure system utilizing electrodes that function as capacitors in nature and as such, has the ability of both storing energy while cleaning water although the purity of fresh water may not withstand that of RO. CDI is in no way a competitive process with RO but augments or complements the deficiency of the latter by performing more effectively for brackish water of low concentration. It is a growing technology that offers large room for improvement especially in the material aspect. Possibly with the development of hybrid materials, computational approach, process control, and modelling strategies, we believe that CDI could be more efficient in high water recovery and energy storage. One interesting perspective of CDI is seeking for selectivity by developing or tailoring electrode physico-chemical properties.

In FCDI, an important research area should be geared towards scaling up of net production of fresh water. Although, continuous flow of operation has been introduced to tackle this challenge yet, it is still difficult to achieve mass production of fresh water because of limited influent (feed solution) flow rate. Current efficiency in FCDI is practically low in comparison to CDI/MCDI due to the water component (weak electrolyte) of the feed electrode thus impeding fast charge transfer from the current collector to the slurry. This is a major challenge, hence introduction of coupling nano-pillars such as Cu or Ni nano-pillars to graphite current collector could improve contact area and offer good electronic conductivity [120,121,122,123].

Poor contact area between current collectors and carbon slurry caused by low level of connectivity of electron network in the carbon slurry is another challenge. Improvement of this area also lies in the material aspect. Research in surface treatment of carbon and synthesis of nano-structured carbon should be encouraged to improve contact and surface area of potential carbon slurry electrode.

However, in FCDI, because of the use of an electrical potential as driving force, membrane fouling propensity is less important. Moreover, Kerwick et al. [122] reported fouling reduction by switching electrodes potential in electrochemical system. Additionally, application of pulse field to electrodes has proven to reduce organic and inorganic fouling. In CDI, organic matters present in water could be adsorbed on the pores of the electrode, thus limiting its adsorption capacity. 

Membrane incorporation in FCDI increases interfacial resistance although, as a proof of concept, Hatzell et al. [97] introduced FCDI without the use of membrane. Additionally, porous ceramic spacer has been recently introduced as IEMs replacement [123] although most FCDI related desalination process still use membrane. Utilization of electro-conductive membranes to improve ion permeation and reduction in resistance can also be a good innovative means of achieving high practicality in FCDI [124].

In FCDI, 35 wt. % of carbon loading has been achieved with high water recovery rate of 70% for a brackish solution of 1000 mg/L and energy consumption lower (by 16–20%) than 1-stage brine water RO unit was reported by Cheol et al. [125]. This innovative process could lead to evolution of future approaches in FCDI sustainability and performance minimizing its drawbacks. FCDI is at its budding stage, knowledge transfer to industrial application has not been confirmed. Research is still at a lab scale, hence the feasibility of FCDI as an alternative to conventional CDI and RO is still debatable. At present, there has been little research in the area of energy recycling of FCDI; a distinctive and fascinating feature of FCDI will be achieved if the energy recovered can be reused to power the cell for additional desalination.

Progress in cell FCDI process and cell configuration has improved its performance, thus yielding better results; though still very young or new, this technology could pave a new dimension as an alternative future of desalination when considering vital aspects like application (for both brackish and sea water), cost, and energy recovery, thus innovation in new architectural FCDI cells and its system performance study can be a future study as the flow electrode system has inherent challenges of clogging.

FCDI is an admixture of factors involving conductivity, viscosity, etc. whose importance is not primarily on adsorption capacity but adsorption rate and stability thus enabling larger room for versatility of flow electrode that could attain this aim.

Hybrid CDI (involving battery electrode) should be an important area of research in the near future especially in terms of hybrid CDI and FCDI combination for sea water desalination at a low energy requirement without attaining high degradation state in performance over long cycles. Whether this innovation will compete effectively with sea water reverse osmosis is a matter of time.

Many challenges still exist (electrode selection compromise, proton or hydroxyl generation, ion co-adsorption, etc.) and the journey to CDI/MCDI/FCDI aim actualization is still far-fetched albeit, predictor designs and models can be introduced i.e., for optimal material design in predicting ion transport in porous materials, for parametric performance to reduce cost and also in robust electrode stability. Table 6 highlights some significant advantages, disadvantages, and differences between CDI/MCDI and FCDI. 

## Figures and Tables

**Table 1:** Progress in innovation in electrochemical desalination [3].

Electrochemical Technology	Innovators	Year
Flow-by CDI	Bair and Murphy	1960
Flow-through CDI	Johnson	1970
MCDI	Lee	2006
Desalination battery	Pasta	2012
Flow electrode CDI	Jeon	2013
Hybrid CDI	Lee	2014
Inverted CDI	Gao	2015
Cation intercalation desalination	Smith and Dimello	2016

**Table 2:** Specification of some selected sea water reverses osmosis (SWRO) desalination plants working in single stage configuration in different countries. Capacity and specific energy consumption (SEC) of reverse osmosis (RO) system are listed as reported by Kim et al. [10].

Country	Plant	Capacity (m^3^.d^−1^)	SEC (kWh.m^−3^)	Reference
Qatar	Ras Abu	164,000	4.5	[11]
Oman	Sur	80,000	3.6	[12]
Spain	Aguilas Gudalentin	200,000	4.6	[13]
Chile	El Coloso	45,360	4.3	-
China	Caofeidian	50,000	4.0	[14]
USA	Carlsbad	190,000	3.5	[14]
Egypt	Marsa	24,000	6.7	[15]
Algeria	Benisaf	200,000	4.0	[16]

**Table 3:** Some recent advances in electrode modifications for capacitive desalination.

Electrode Material	Water Salinity (ppm NaCl)	Capacity (mg/g)	Operation Voltage (V)	Ref.
NG-CNFs	1000	14.79	1.2	[61]
Graphene gel	500	49.34	2.0	[62]
3D Graphene modified with SWCNT	300	48.73	2.0	[63]
GO/resorcinol formaldehyde microsphere (GORFM)	800	35.52	1.8	[64]
rGO/TiO_2_	300	9.1	0.8	[65]
ZrO_2_/GO	50	4.55	0.8–1.2	[66]
PANI/AC	250	3.15	1.2	[67]
Flexible Graphene	300	18.43	1.4	[68]
3D Graphene grafted with amine-sulfonic functional group	500	13.72	1.4	[69]
GO-CNT/AC	800	21.3	0.3–1.5	[70]

**Table 4:** Mains advantages and drawbacks of flow-by and flow-through CDI cells for desalination.

CDI Geometry	Advantages	Drawbacks
Flow-by	-Easy to design with low cost plate dense electrodes -High energy recovery -Low energy consumption	-Moderate water flow rate due to low electrodes interspace -Long times required for desalination due to quite low kinetics
Flow-through	-High ASAR due to high kinetics promoted by turbulence inside the pores of electrodes -High mSAC of the porous electrodes -High water flow rate due to low fluidic resistance of aerogel electrodes	-Higher CAPEX due to the cost of porous electrodes

**Table 5:** Summary of some recent advances in operating parameters for flow capacitive deionization systems as found in literature.

Slurry Electrodes				Feed Solution			
AC (Wt %)	[NaCl] (mM)	Additive (Wt %)	Flow Rate (mL/min)	[NaCl] in Effluent (mM)	Flow Rate (mL/min)	Voltage (V)	Ref.
0–16	60	-	0–175	60	2–13	1.2	[91]
20	11.1	CB 0.5-1.5	4	-	11	0.6–4.8	[92]
5	0.1	-	1	-	1	1.2	[93]
5	2.5	FCNT 0.25	25	-	3	1.2	[94]
10	0.011–0.016	CNT 1.5	15	9.01	15	-	[95]
5–35	2.5	-	25	3.5	3	1.2	[96]
5	-	-	60	17.1–256.4	9	1.2	[97]
5	1.7	-	1	3.42–598.3	1	1.2	[102]
5–23	10	-	5	5	-	-	[103]
8.3	20	-	0.5–1.5	20	0.5–1.5	1–1.91	[104]
9.1	100			-	-	1.2	[105]
25	17.1	-	-	-	-	1.2	[106]
25	2.5	-	-	3.5	-	1.2	[107]

**Table 6:** Pros and cons with major differences between CDI/MCDI/FCDI.

Technology	Advantages	Disadvantages	Major Differences
CDI	Easy to set up Low resistance Low capital cost	Ion co-adsorption Low charge efficiency Low mSAC Low selectivity Low ASAR Electrodes are fixed in solid state hence limited surface area for pores ion adsorption	Does not involve ion exchange membrane.
MCDI	High kinetics High charge efficiency High ASAR High mSAC Membrane tunability is possible in case of multi-ion system thus selectivity can be achieved.	High resistance High energy demand Electrodes are fixed in solid state hence limited surface area for pores ion adsorption	It uses ion exchange membrane
FCDI	Binder free hence ion adsorption is improved. Capacitance is enhanced as uncharged carbon particles are continuously fed into the charging cell Could be membrane free. It allows continuous or infinite ion adsorption Applicable for sea water desalination	Low electron within carbon network. Low charge transfer Low flow rate of feed solution.	It involves different architectural design. Electrode is in liquid and continuous flowing state and not fixed on current collectors.

## Abbreviations

CEM	Cation exchange membrane
AEM	Anion exchange membrane
SWCNT	Single wall carbon nanotube
GO	Graphene oxide
PANI	Polyaniline
FGraphene	Functionalized grapheme
FCNT	Functionalized carbon nanotube
3DGR	3-dimensional graphene
NG-CNFs	Nitrogen doped carbon nanofibers
